# Cell-Permeable Bak BH3 Peptide Induces Chemosensitization of Hematologic Malignant Cells

**DOI:** 10.1155/2020/2679046

**Published:** 2020-11-30

**Authors:** Omar Ugarte-Alvarez, Paola Muñoz-López, Liliana Marisol Moreno-Vargas, Diego Prada-Gracia, Armando Alfredo Mateos-Chávez, Elayne Irene Becerra-Báez, Rosendo Luria-Pérez

**Affiliations:** ^1^Unit of Investigative Research on Oncological Diseases, Children's Hospital of Mexico Federico Gomez, Mexico City 06720, Mexico; ^2^Posgrado en Biomedicina y Biotecnología Molecular, Escuela Nacional de Ciencias Biológicas, Instituto Politécnico Nacional, Mexico City 11340, Mexico; ^3^Research Unit on Computational Biology and Drug Design, Children's Hospital of Mexico Federico Gomez, Mexico City 06720, Mexico

## Abstract

Hematologic malignancies such as leukemias and lymphomas are among the leading causes of pediatric cancer death worldwide, and although survival rates have improved with conventional treatments, the development of drug-resistant cancer cells may lead to patient relapse and limited possibilities of a cure. Drug-resistant cancer cells in these hematologic neoplasms are induced by overexpression of the antiapoptotic B-cell lymphoma 2 (Bcl-2) protein families, such as Bcl-_XL_, Bcl-2, and Mcl-1. We have previously shown that peptides from the BH3 domain of the proapoptotic Bax protein that also belongs to the Bcl-2 family may antagonize the antiapoptotic activity of the Bcl-2 family proteins, restore apoptosis, and induce chemosensitization of tumor cells. Furthermore, cell-permeable Bax BH3 peptides also elicit antitumor activity and extend survival in a murine xenograft model of human B non-Hodgkin's lymphoma. However, the activity of the BH3 peptides of the proapoptotic Bak protein of the Bcl-2 family against these hematologic malignant cells requires further characterization. In this study, we report the ability of the cell-permeable Bak BH3 peptide to restore apoptosis and induce chemosensitization of acute lymphoblastic leukemia and non-Hodgkin's lymphoma cell lines, and this event is enhanced with the coadministration of cell-permeable Bax BH3 peptide and represents an attractive approach to improve the patient outcomes with relapsed or refractory hematological malignant cells.

## 1. Introduction

Hematologic malignancies such as acute lymphoblastic leukemia (ALL) and non-Hodgkin's lymphoma (NHL) are among the main causes of death in pediatric cancer patients throughout the world [1, 2]. These neoplasms arise from the transformation of immature cells in the case of leukemias and of immature or mature cells in lymphomas, mainly compromising the B lymphocyte population and in a lesser proportion that of T lymphocytes and NK cells [3]. Fortunately, conventional treatments such as chemotherapy and radiotherapy have increased 5-year survival to 92% in pediatric patients with acute lymphoblastic leukemias and 91% in pediatric patients with non-Hodgkin's lymphoma [4–6]. However, drug resistance hinders the complete success of these therapies [7]; so, new antitumor therapies must be sought that can completely eradicate drug-resistant transformed cells [8].

Among the mechanisms implicated in cancer drug resistance, there are abnormalities in the genes and proteins of the B-cell lymphoma 2 (Bcl-2) family [9, 10] that control the apoptosis mitochondrial pathway. In cells, the balance between death and survival is controlled by members of three groups of this family of proteins: the group of multidomain antiapoptotic BH1-4 proteins (Bcl-2, Bcl-_XL_, Bcl-w, Mcl-1, and A1) that promote cell survival by inhibiting proapoptotic multidomain proteins; the group of multidomain proapoptotic proteins BH1-3 (BH1-3) (Bax, Bak, and Bok), which are apoptosis effectors; and the group of proapoptotic BH3-only proteins (Bid, Bim, Puma, Noxa, Bad, Bmf, Hrk, and Bik) that initiate apoptosis [11–13]. In healthy cells, antiapoptotic proteins bind and inhibit the Bax or Bak effector proteins by blocking their polymerization on the mitochondrial surface and preventing the initiation of apoptosis [11–14]. BH3-only proteins are induced in response to stress signals and promote apoptosis by directly binding to effector proteins or binding to antiapoptotic proteins to release the effector proteins [15]. In this balance, overexpression of antiapoptotic proteins in tumor cells promotes survival of the transformed cell and represents a mechanism of resistance to treatment [16]. Overexpression of antiapoptotic proteins such as Bcl-2, Bcl-_XL_, and Mcl-1 has found to be associated with drug resistance in human tumor cell lines [17–19], including leukemia [17] and NHL cells [20–23]. The interaction of proteins of the Bcl-2 family through a hydrophobic groove formed by its BH domains [11–13] has been key in reverting this resistance mechanism.

Peptides derived from the BH3 domain of proapoptotic proteins have been shown to bind to antiapoptotic proteins, thus antagonizing their function [24–26]. In this context, the use of peptides derived from the BH3 domain of the Bax and Bad proteins antagonized the activity of the antiapoptotic proteins, Bcl-2 and Bcl-_XL_, and induced the release of cytochrome c from the mitochondria of cells in T-cell acute leukemia [27]; furthermore, when binding the BH3 domain peptides of the Bax, Bad, and Bak proteins to the fusogenic peptide of the Antennapedia protein (cell-permeable BH3 peptides) to make them permeable to tumor squamous cells in carcinoma of the head and neck and in T-cell acute leukemia cells, they blocked the activity of Bcl-_XL_ and Bcl-2, restoring apoptosis [28]. We have previously reported that bactofection of plasmids encoding a peptide from the BH3 domain of the proapoptotic Bax protein, antagonized the antiapoptotic activity of the Bcl-2 family proteins, restored apoptosis, and induced chemosensitization of tumor cells [29], and we have recently documented that a cell-permeable Bax BH3 peptide expressed and released into the tumor microenvironment via a live-attenuated bacterial vector promoted apoptosis, induced antitumor activity, and increased survival in a murine xenograft model of human B non-Hodgkin's lymphoma [30]. However, the activity of the BH3 peptides of the proapoptotic Bak protein of the Bcl-2 family against these hematologic malignant cells requires further characterization. In this study, we report the ability of the Bak BH3 peptide coupled with the Antennapedia fusogenic peptide (cell-permeable Bak BH3 peptide) to promote apoptosis and induce chemosensitization of acute lymphoblastic leukemia and non-Hodgkin lymphoma cell lines.

## 2. Materials and Methods

### 2.1. Molecular Modeling by Homology

To generate the model of the cell-permeable Bak BH3 peptide, APTK chimera (Antennapedia fusogenic peptide = AP, Flag peptide = T, and Bak BH3 peptide = K), we used two independent strategies and then chose the consensus model. On the one hand, we used an assembly of large rigid fragments obtained from similar structures aligned by means of their primary and secondary sequences. This methodology cuts and pastes fragments of the peptide skeleton of known structures (SWISS-MODEL) [31, 32]. On the other hand, we used modeling satisfying the molecular constraints extracted from databases and similarly aligned structures. This method helps produce a set of structures for the A sequence, all of which are compatible with the restrictions observed in the templates (MODELLER) [33, 34].

### 2.2. Geometry Optimization of the Proposed Models

Once the 3D models were prepared, hydrogen atoms were added, and side-chain orientations were optimized through the energy minimization steepest descent method, using the CHARMM36 force field [35] in a TIP3P water box [36] with the molecular dynamics engine OpenMM [37].

### 2.3. Stereochemical Quality Evaluation of the Models

Coordinate files of the 3D models were sent to MolProbity [38] to produce a Ramachandran plot (*ϕ* and *ψ* angles) reflecting polypeptide chain distortion in the nonfully allowed region. We also sent the coordinate files to RAMPAGE to identify side chains with less common conformations possibly because of local protein tension [39]. The quality of the models was further validated using two additional tools: ProQ3/ProQ3D [40, 41] and QMEAN [42].

### 2.4. Peptides and Drugs

The peptide sequences used in our study are as follows: for the BH3 domain peptide of the Bak protein (K), we used the sequence MGQVGRQLAIIGDDINRRY [28], for the BH3 domain peptide of the Bax protein, we used the sequence STKKLSECLKRIGDELDSN (X) [30], for the Flag peptide, we used the sequence DYKDDDDK (T) [43], and for the Antennapedia fusogenic peptide, we used the sequence RQIKIWFQNRRMKWKK (PE), also known as Penetratin [28]. The peptides used in this study were synthesized by Peptide 2.0 Company, USA. The negative control peptides were PET = RQIKIWFQNRRMKWKKDYKDDDDK and KT = MGQVGRQLAIIGDDINRRYDYKDDDDK and the peptide complexes of the cell-permeable Bak peptide PETK = RQIKIWFQNRRMKWKKDYKDDDDKMGQVGRQLAIIGDDINRRY and cell-permeable Bax peptide PEX = RQIKIWFQNRRMKWKKSTKKLSECLKRIGDELDSNM. All peptides were diluted in sterile water. The drug cisplatin (CDDP, cis*-*diamino-dichloro-platin II) from Sigma-Aldrich was diluted in dimethylsulfoxide (DMSO). Vincristine (Sigma-Aldrich) was diluted in sterile water.

### 2.5. Cell Lines and Cell Cultures

The Ramos RA1 cell line (Burkitt's lymphoma), a human B non-Hodgkin's lymphoma (ATCC, CRL-1596), and a CCRF-CEM cell line of human T acute lymphoblastic leukemia (ATCC, CCL-119) were obtained from the American Type Cell Collection and cultured in advanced RPMI 1640 medium (Invitrogen), supplemented with 1% antibiotics-antimycotics, with 10000 U/mL penicillin G, 10 mg/mL streptomycin, 25 *μ*g/mL amphotericin B, and 4% fetal bovine serum (FBS, Invitrogen). Cultures were permanently maintained at 37°C and 5% CO_2_. For the infection assays, the cells were grown with advanced RPMI 1640 medium (Invitrogen) and supplemented with 2% FBS without antibiotics (Invitrogen). Peripheral blood mononuclear cells (PBMC) from healthy donors were obtained from the blood bank of the Children's Hospital of Mexico Federico Gomez, isolated by Ficoll-Paque Plus (GE Healthcare Life Sciences), and cultured under Ramos cells conditions.

### 2.6. Detection of the Antiapoptotic and Proapoptotic Molecules by Western Blot

Detection of the antiapoptotic and proapoptotic molecules was conducted with slight modifications to the technique described by Mateos-Chavez et al. [30]. A million Ramos or CCRF-CEM cells were lyzed with RIPA lysis buffer (Sigma-Aldrich) supplemented with a cocktail of protease inhibitors (Roche). Protein quantification was performed with the bicinchoninic acid kit by Thermo Fisher Scientific. For the Western blot, 40 *μ*g of total protein was placed in each well, and electrophoresis on 15% polyacrylamide-SDS gel was performed. The proteins were then transferred to nitrocellulose membranes (Bio-Rad) with a Trans-Turbo Blot System by Bio-Rad (25 volts, 10 minutes). For the detection of the Bcl-_XL_, Mcl-1, Bcl-2, Bak, and Bax proteins, we used anti-Bcl-_XL_, anti-Mcl-1, anti-Bak, and anti-Bax antibodies (cell signaling) induced in rabbits and anti-Bcl-2 (cell signaling) induced in mouse, all of them diluted 1 : 500 in blocking buffer (Li-Cor), and as the secondary antibody, goat anti-rabbit IgG IR Dye 800cw and goat anti-mouse 680cw (Li-Cor) diluted 1 : 10000 in blocking buffer (Li-Cor). As a constitutive protein control, we used an anti-*β*-tubulin antibody (Abcam), induced in rabbit and diluted at 1 : 8000 in blocking buffer (Li-Cor). Finally, the image was obtained and analyzed in the system for infrared fluorescent imaging, Odyssey CLx (Li-Cor).

### 2.7. Treatment of Ramos and CCRF-CEM Cells with the Peptides and/or the Drug Cisplatin

The Ramos and CCRF-CEM were treated with 20 *μ*M [44] of the different peptides for 12 hours, in the presence or absence of the drug cisplatin (CDDP) 40 *μ*M. The cells were then subjected to various protocols to evaluate cellular viability and apoptosis.

### 2.8. Cell Viability Assay

Twenty thousand Ramos and CCRF-CEM cells were cultured in 96-well plates with 20 *μ*M of the PETK, PET, and KT peptides, with or without cisplatin, 40 *μ*M. After 12 hours of treatment, the cells were processed to determine viability with the MTT (3-(4, 5-dimethylthiazol-2)-2, 5-diphenyltetrazolium bromide)), according to the manufacturer's instructions (Roche). Briefly, 10 *μ*L of the MTT reagent was added to the wells with treated and untreated cells; they were then incubated for 4 hours at 37°C and 5% CO_2_. Subsequently, 100 *μ*L of the solubilizing reagent was added to each well, and these were incubated overnight at 37°C and 5% CO_2_. Finally, they were read at 550 nm in an EnSpire plate multireader (Perkin Elmer).

### 2.9. Evaluation of Caspase 3 Activity by Flow Cytometry

These assays were conducted with modifications to the protocol described by Mateos-Chávez et al. [30]. After treatment of the Ramos and CCRF-CEM cells (200,000 cells) with the different peptides at 20 *μ*M, with or without cisplatin 40 *μ*M, cells were washed, fixed, and permeabilized for intracellular staining with active anti-caspase-3 antibody-FITC (BD Pharmingen). Data collection and analysis were conducted in a CytoFLEX (Beckman Coulter) cytometer.

### 2.10. Statistical Analysis

To determine the differences between cell groups treated with the different peptides, we used one-way analysis of variance (ANOVA) and post hoc Bonferroni tests, with a 95% confidence interval. In all cases, the average of three or more independent experiments is presented ± the standard deviation (SD). Differences were considered significant at *p* values ≤ 0.05 in all comparisons. Statistical analysis was performed with GraphPad Prism 6 and IBM SPSS Statistics software.

## 3. Results

### 3.1. 3D Structure of the Cell-Permeable Bak BH3 Peptide

**Figure 1:**
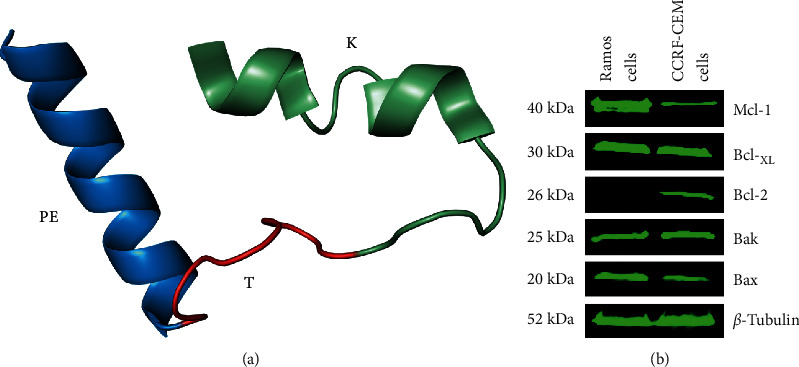
3D structure of cell-permeable Bak BH3 peptide (PETK) and expression of the Bcl-2 family proteins in hematologic malignant cells. (a) The structure is characterized by having two *α*-helices connected by a loop. The folding and packing allow the visualization of a structural domain of the bHLH type: a minor helix (Bak BH3 peptide = K) that folds towards the major basic helix (Antennapedia fusogenic peptide = PE) due to the flexibility of the loop (Tag peptide = T); its strong negative charge directs the packaging of the polypeptide. (b) Expression of the antiapoptotic proteins (Mcl-1, Bcl-_XL_, and Bcl-2) and proapoptotic proteins (Bak and Bax) in Ramos (NHL) and CCRF-CEM (ALL) cells. Proteic extracts of hematologic malignant cells (40 *μ*g per well) were analyzed by Western Blot using the anti-Mcl-1, anti-Bcl-_XL_, anti-Bcl-2, anti-Bak, anti-Bax, and anti-*β*-tubulin antibodies. Goat anti-rabbit IgG antibody (IRDye® Odyssey) was used as a secondary antibody.

In order to predict the folded and partially misfolded regions of the cell-permeable Bak BH3 peptide, as well as to contribute to a better description, a molecular modeling and geometry optimization of the tridimensional structure of the cell-permeable Bak BH3 peptide (PETK) was conducted with chemoinformatic techniques. The three-dimensional model is characterized by having two *α*-helices connected by a loop. In general, a helix is smaller (Bak BH3 peptide = K), and due to the flexibility of the loop (Tag peptide = T), it allows the Bak BH3 peptide to dimerize with the larger helix, Antennapedia fusogenic peptide (PE) (Figure 1). The folding and packing allow the visualization of a structural domain of the bHLH type. Stereochemical quality evaluation revealed that 99.8% of the residues are in zones that correspond to nuclear regions, representing physically accessed conformations to *α*-helices. As a result of this evaluation, we were able to determine that only one residue in the model (Ile35) is out of the fully allowed areas of the Ramachandran plot. When performing a theoretical prediction on the internalization of the core in the membrane, we observed that given its amino acid composition and its hydrophobicity, it is possible that it crosses the membrane through a diffusion process (data not shown).

### 3.2. Cell-Permeable Bak BH3 Peptide Induces Apoptosis-Mediated Cell Death in Hematologic Malignant Cell Lines

**Figure 2:**
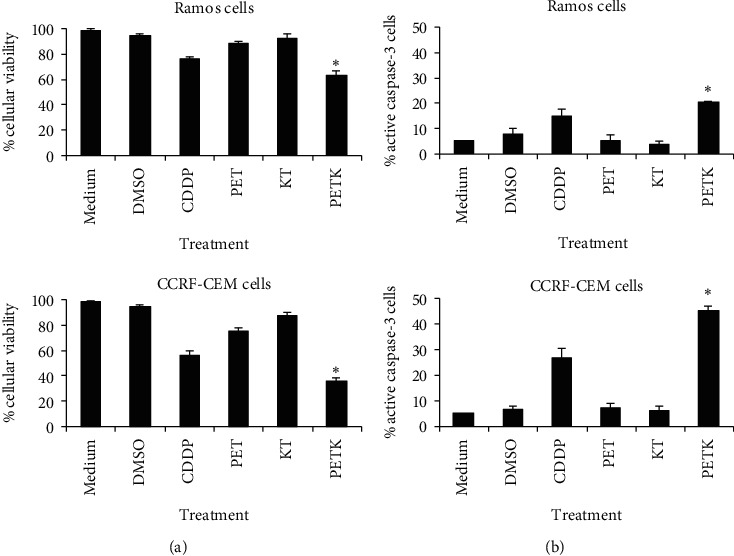
The cell-permeable Bak BH3 peptide decreases the viability and increases apoptosis in hematological malignant cell lines. (a) Cell viability assays. Ramos and CCRF-CEM cells were incubated for 12 hrs with 20 *μ*M of the PET, KT, and PETK peptides. Cisplatin (CDDP) 40 *μ*M was used as a positive control. Viability was analyzed with MTT. Bar graphs represent the average ± SD of three independent experiments. An ANOVA test was conducted with a post hoc Bonferroni test to calculate the *p* value between the study groups. A significant difference was observed between the cells treated with the PETK peptide compared with the other controls (^*∗*^*p* *<* 0.05). (b) Apoptosis assays. Ramos and CCRF-CEM cells were incubated for 12 hrs with 20 *μ*M of the PET, KT, and PETK peptides, using cisplatin 40 *μ*M as a positive control. Apoptosis was quantified with an active caspase-3 assay and analyzed by flow cytometry. The graphs represent the values obtained in three independent assays. An ANOVA test was performed with a post hoc Bonferroni test to establish differences between groups. ^*∗*^*p* *<* 0.05.

To analyze the ability of the cell-permeable Bak BH3 peptide to induce cell death by apoptosis in malignant hematologic cell lines, we conducted cell viability assays and active caspase-3 determinations. Thus, Ramos and CCRF-CEM cells expressing the antiapoptotic proteins Mcl-1, Bcl-_XL_, and Bcl-2 and the proapoptotic proteins Bax and Bak (Figure 1(b)) were treated with the peptides PET, KT, and PETK for 12 h at a concentration of 20 *μ*M and evaluated with the MTT cell viability assay. Untreated cells (medium) and cells treated with the vehicle (DMSO), in which CDDP is dissolved, were used as negative controls, and the positive control was cisplatin (CDDP, a drug used in some cases of refractory non-Hodgkin's lymphoma) [45, 46].  Figure 2(a) shows a significant decrease in Ramos cell viability (NHL) after treatment with our peptide of interest, PETK (62.43% ± 2.5). The CDDP-positive control decreased their viability to average values of 76.4% ± 1, while the control peptides PET and KT also showed a slight decrease in viability to values of 87.7% ± 0.96 and 92.23% ± 2, respectively. Surprisingly, a greater effect was observed in CCRF-CEM (ALL) cells in terms of viability, after treatment with the PETK peptide (36.4% ± 2), almost 26% more than that observed in Ramos cells. Cells treated with CDDP decreased their viability to values of 55.96% ± 2.61, close to 15% more than in Ramos cells, suggesting that CCRF-CEM cells are more sensitive to chemotherapeutic treatment and even to the PETK peptide.

Subsequently, we examined whether the decrease in cell viability was mediated by the induction of apoptosis. For this purpose, Ramos and CCRF-CEM cells were treated with the different peptides at 20 *μ*M for 12 hours and subjected to apoptosis assays such as active caspase-3. As positive controls, cells were treated with 40 *μ*M CDDP, and as negative controls, untreated cells (medium) and cells with DMSO were used.  Figure 2(b) shows that Ramos cells treated with the PETK peptide had a greater number of active caspase-3 cells (20.25% ± 0.3) compared with the other treatments. In this assay, cells treated with CDDP at 40 *μ*M were positive for active caspase-3 in 14.9% ± 2.7, while the PET (5.2% ± 2.0) and KT (3.5% ± 1.3) peptides have very similar baseline values to untreated cells suspended only in medium (5.0% ± 0.0). In the case of CCRF-CEM cells (Figure 2(b)) treated with the PETK peptide, there was a greater number of active caspase-3 positive cells (45.6% ± 1.1) compared with untreated cells (5.0% ± 0.0), while in cells treated with PET (7.1% ± 1.5) and KT (6.2% ± 1.5), there were no significant differences compared with untreated cells. In the group treated with CDDP as a positive control, we obtained only 26.9% ± 3.6 of active caspase-3 positive cells. These results show that as in Ramos cells, the CCRF-CEM treated with the PETK peptide significantly increased the apoptosis when compared with the cells treated with the chemotherapy drug CDDP and other controls. It is important to mention that these data confirm that CCRF-CEM cells are more sensitive than Ramos cells to treatment with the PETK peptide and the drug CDDP.

### 3.3. Cell-Permeable Bak BH3 Peptide Induces Chemosensitization of Hematologic Malignant Cell Lines

**Figure 3:**
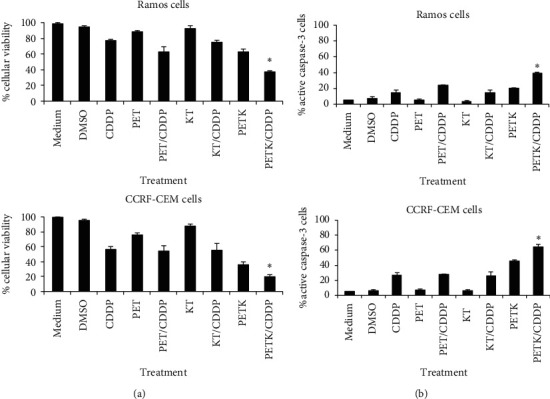
Sensitivity to chemotherapy mediated by the cell-permeable Bak BH3 peptide. Ramos and CCRF-CEM cells were incubated for 12 hrs with 20 *μ*M of the PEKT, PET, and KT peptides, in the presence or absence of cisplatin (CDDP) 40 *μ*M. (a) Cell viability was analyzed with MTT assays. (b) Apoptosis was quantified with the active caspase-3 assay and analyzed by flow cytometry. In both assays, the graphs represent the values of three independent assays. An ANOVA test was performed with Bonferroni post hoc analysis to establish the difference between groups. ^*∗*^*p* *<* 0.05.

Treatment failure in patients with hematologic malignancies is not only mediated by drug resistance but is also associated with drug toxicity; solving this problem is a great challenge in the clinic. One of the strategies to damper this issue is the subtoxic administration of drug doses in conjunction with molecules that sensitize tumor cells to die by apoptosis. Hence, we analyzed the ability of the cell-permeable Bak BH3 peptide (PETK) to induce sensitization to chemotherapy, particularly to cisplatin (CDDP), a drug used in the management of some cases of refractory hematologic malignancies [45, 46]. Considering that the experimentally determined toxic dose 50 (TD50) of CDDP in Ramos cells was 167 *μ*M and 37 *μ*M in CCRF-CEM cells after 12 hours of treatment (data not shown), and we used 40 *μ*M of this drug as a subtoxic dose to analyze the sensitivity to chemotherapy induced by the cell-permeable Bak BH3 peptide. We, therefore, treated Ramos and CCRF-CEM cells for 12 hrs with the different peptides, at 20 *μ*M, with or without cisplatin 40 *μ*M and then performed cell viability assays by MTT and apoptosis by active caspase-3. As shown in Figure 3(a), the Ramos cells treated with the cell-permeable Bak BH3 peptide in the presence of CDDP, considerably decreased in terms of cellular viability to 37.3% ± 1.4, which represents 25% less viability than in cells that were only treated with the PETK peptide (62.4% ± 3.6), and 40% less than that of cells that were only treated with CDDP (76.4% ± 1.4). In the case of CCRF-CEM cells treated with the cell-permeable Bax BH3 peptide and CDDP, cell viability also decreased considerably to 20.2% ± 1.8, 16% less viability than cells that were only treated with the PETK peptide (36.42% ± 2.8), and 35% less than the cells treated with only CDDP (55.99% ± 3.7). The controls, medium, DMSO, PET, and KT peptides had baseline cell viability values above 90%.

To analyze whether this increase in sensitivity to chemotherapy was mediated by an increase in apoptosis, we determined the percentage of active caspase-3 positive Ramos and CCRF-CEM cells treated with the peptides in the presence or absence of CDDP.  Figure 3(b) shows a clear increase in the percentage of active caspase-3 positive Ramos cells after treatment with the PEXT peptide and cisplatin (39.252 ± 1.3), compared with those that were only treated with the PEXT peptide (20.25 ± 0.3), or only with cisplatin (14.9 ± 2.7). Cells treated with the controls, medium, DMSO, and the PET and KT peptides established the baseline apoptosis values. Data obtained with the CCRF-CEM cells also confirmed an increase in apoptosis after treatment with the PEKT peptide in the presence of cisplatin (64.8% ± 3.11), in comparison with the cells that were only treated with PEKT (45.6% ± 1.5) or CDDP (26.96% ± 3.6). Cells treated with the controls, medium, DMSO, PET and KT peptides established the apoptosis baseline values. These results confirmed the ability of the cell-permeable Bak BH3 peptide to chemosensitize hematologic malignancy cells.

### 3.4. Cell-Permeable BH3 Peptides from Proapoptotic Bak and Bax Proteins Increase Cell Death in Hematologic Malignant Cell Lines

**Figure 4:**
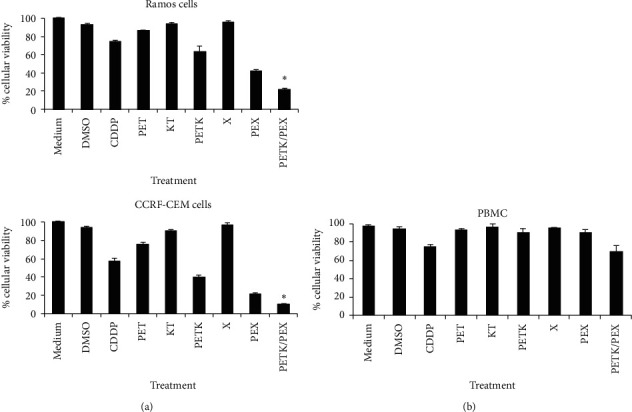
The combination of cell-permeable Bak and Bax BH3 peptides increases cell death of hematologic malignant cell lines. (a) Ramos and CCRF-CEM cells were incubated for 12 hrs with 20 *μ*M of the PETK, PET, KT, X, and PEX peptides and the PETK and PEX combination. CDDP 40 *μ*M was used as a positive control. (b) Peripheral blood mononuclear cells (PBMC) were incubated for 12 hrs with 20 *μ*M of the PETK, PET, KT, X, and PEX peptides and with the PETK and PEX combination. CDDP 40 *μ*M was used as a positive control. In both cases, cell viability was determined with MTT assays. The graphs represent the value of three independent assays. The ANOVA test was performed with Bonferroni post hoc analysis to determine the difference between groups. ^*∗*^*p* *<* 0.05.

Considering the synergistic effect on death induction in tumor cells after the administration of two BH3 peptides from proapoptotic proteins or two synthetic BH3 mimetics [47–49], we established whether the combination of the cell-permeable Bak BH3 peptide (PETK) and the cell-permeable Bax BH3 peptide (PEX) could increase death in malignant hematologic cells. Thus, Ramos and CCRF-CEM cells were treated with 20 *μ*M of the different peptides and incubated for 12 hours.  Figure 4(a) reveals the significant decrease in Ramos cell viability after treatment with both PETK and PEX peptides, with values of 21.84 ± 1.4, reflecting 41% less viability than in cells that were only treated with the PETK peptide (63.49% ± 6.0), 20% less viability than the cells that were only treated with the PEX peptide (42.37% ± 0.8), or 51% less when compared with the positive CDDP control (74.33% ± 1.1). In CCRF-CEM cells, we observed a more dramatic effect in the decrease in cell viability after treatment with the combination of PETK and PEX peptides (9.93 ± 1.2), a value equivalent to 30% less cellular viability than that observed in the cells treated with PETK (40.00% ± 1.5), 12% less viability than cells treated with PEX (21.98% ± 0.9), or 47% less viability than the positive CDDP control (57.30% ± 2.6).

In order to analyze the toxicity of the peptides used in this study on normal cells, peripheral blood mononuclear cells (PBMC) were treated with the different peptides for 12 hours and analyzed for cell viability assays with MTT; results showed a slight decrease in cell viability, with over 90% of live cells after treatment with the PEXT, PET, KT, X, and PEX peptides (Figure 4(b)). Overall, these results confirm that the combination of cell-permeable Bak BH3 peptide and the cell-permeable Bax BH3 peptide have a synergistic effect on the induction of cell death in hematologic malignant cell lines and that the peptides described in the study do not mediate a cytotoxic effect on normal cells such as PBMC.

### 3.5. Enhanced Chemosensitization of Hematologic Malignant Cell Lines Mediated by the Combination of Cell-Permeable Bak and Bax BH3 Peptides

Once the ability of the combination of cell-permeable Bak and Bax BH3 peptides was shown to promote death in 78% of Ramos cells and 90% of CCRF-CM cells, better than the chemosensitization efficacy of the hematologic malignancies in the presence of the cell-permeable Bak BH3 peptide and cisplatin, we analyzed whether the combination of these peptides further increased sensitization to chemotherapy in hematologic malignant cells.

**Figure 5:**
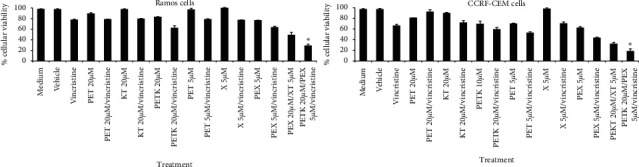
The combination of cell-permeable BH3 peptides enhances chemotherapy sensitivity in hematologic malignant cell lines. Ramos and CCRF-CEM cells were incubated for 6 hrs with 20 *μ*M of the PEKT, PET, and KT peptides and 5 *μ*M of the X and PEX peptides and the combination of PEXT and PEX. Vincristine 80 nM was the chemotherapeutic agent. In both cases, cell viability was determined with MTT assays. The graphs represent the values of three independent assays. The ANOVA test was performed with Bonferroni post hoc analysis to establish the difference between groups. ^*∗*^*p* < 0.05.

Thus, Ramos and CCRF-CEM cells were treated with the different peptides and the combination of the cell-permeable Bak BH3 peptide (PETK) plus the cell-permeable Bax BH3 peptide (PEX), in the presence of a subtoxic dose of vincristine, a commonly used drug in the management of hematologic malignancies [50]. The experimentally determined toxic dose 50 (TD50) of vincristine in Ramos cells was 280 nM and 177 nM in CCRF-CEM cells (data not shown). Based on these results, Ramos and CCRF-CEM cells were treated for 6 hr with the PET, KT, and PETK peptides at 20 *μ*M and with the X and PEX peptides at 5 *μ*M (the dose of these peptides was decreased to underscore the synergistic effect in inducing tumor cell death), in the presence or absence of the subtoxic dose of vincristine, 80 nM; they were then tested for cell viability by MTT.  Figure 5 shows that the Ramos cells had a slight decrease in cell viability in response to vincristine treatment (76.18% ± 1.6), cell-permeable Bak BH3 peptide (82.80% ± 1.0), or the cell-permeable Bax BH3 peptide (76.42% ± 0.7), and that treatment with both cell-permeable BH3 peptides (PETK and PEX) significantly decreased cell viability to values of 48.5% ± 5.4, that further decreased by 20% in the presence of vincristine (28.5% ± 2.9). It is important to mention that the cells treated with the PETK (61.0 ± 4.4) or PEX (63.31 ± 2.0) peptides in the presence of vincristine only had a moderate decrease in cell viability, as expected.

In CCRF-CEM cells, we observed a moderate decrease in cell viability after treatment with vincristine (66.17% ± 2.7), cell-permeable Bak BH3 peptide (69.10% ± 5.7), or the cell-permeable Bax BH3 peptide (62.13% ± 0.7), and treatment with both cell-permeable BH3 peptides (PETK and PEX) significantly decreased cell viability to values of 32.02% ± 2.8. This decrease dramatically increased by 14% in the presence of vincristine (18.4% ± 4.4). Cells treated with the PETK (59.26 ± 3.4) or PEX (43.19 ± 1.5) peptides in the presence of vincristine showed a moderate decrease in cell viability, as expected. Overall, these results show that treatment with the combination of the cell-permeable Bak and Bax BH3 peptides increases chemosensitization in hematologic malignant cells.

## 4. Discussion

Worldwide, hematologic malignances are among the first causes of death in the pediatric population, although treatment with chemotherapy multiagents has increased survival to 5 years in non-Hodgkin's lymphoma (NHL) in 85% of cases [5, 6], and in 95% of cases of acute lymphoblastic leukemia (ALL) [51], close to 20% of the population with these neoplasms fails treatment, relapse, and dies [51]. This less than favorable scenario has led to the research and development of new antitumor therapies that can completely eradicate drug-resistant transformed cells [8]. Among the causes of treatment failure, drug resistance is pivotal [7] and can be mediated by the dysregulation of genes and proteins of the Bcl-2 family that play a key role in intrinsic apoptosis [9, 10]. In healthy cells, antiapoptotic proteins from the Bcl-2 families, such as Bcl-2, Bcl-_XL_, and Mcl-1 among others, bind to and inhibit the effector proteins Bax or Bak, blocking their polymerization on the mitochondrial surface and preventing the initiation of apoptosis [14]. Based on this required balance, the overexpression of antiapoptotic proteins in tumor cells promotes the survival of the transformed cell and represents a mechanism of treatment resistance [16]. In NHL and ALL, overexpression of the antiapoptotic proteins Bcl-2 [20, 21, 52], Bcl-_XL_ [22, 23, 52], and Mcl-1 [52, 53] have been documented to be associated with drug resistance. Reverting this resistance mechanism has been possible due to the structural studies that reveal that proteins from the Bcl-2 family interact between themselves via a hydrophobic groove formed by its BH domains [11–13]; in this way, BH3 domain-derived peptides from proapoptotic proteins can bind to the antiapoptotic proteins and antagonize their function [24–26].

On this basis, BH3 domain hydrophobic peptides of the Bax, Bad, and Bak proteins once coupled to the Antennapedia fusogenic peptide (cell-permeable Bax BH3 peptides) to make them permeable to squamous cell carcinoma tumor cells and T-cell acute leukemia cells, blocking the activity of Bcl-_XL_ and Bcl-2 and restoring apoptosis [28]. Other studies have documented that the use of peptides from the BH3 region of the BIM protein is effective in the treatment of cell lines from refractory hematologic malignancies [54], and the use of small molecules that mimic the function of the BH3-only proteins in the treatment of refractory hematologic malignancies has been also explored [55], as in the case of ABT-737 [56, 57] and its orally bioavailable derivatives, ABT-263/navitoclax [58] (both bind with high affinity to Bcl-2, Bcl-_XL_, and Bcl-w), GX15-070/obatoclax [59] (an inhibitor targeting all antiapoptotic Bcl-2 family proteins), and ABT-199/venetoclax (a Bcl-2 antagonist), and this last was recently approved by the FDA for the treatment of chronic lymphocytic leukemia (CLL) but not of non-Hodgkin's Lymphoma [15, 44]. We have previously reported that the bactofection of sequences encoding a peptide from the BH3 domain of the proapoptotic Bax protein, antagonized the antiapoptotic activity of the Bcl-2 family proteins, restored apoptosis, and induced chemosensitization of tumor cells [29]; also, we recently reported that a cell-permeable Bax BH3 peptide expressed and released into the tumor microenvironment via a live-attenuated bacterial vector, promoted apoptosis, induced antitumor activity, and increased survival in a murine xenograft model of human B non-Hodgkin's lymphoma [30]. However, the activity of the BH3 peptides from the proapoptotic Bak protein of the Bcl-2 family against hematologic malignant cells and particularly their ability to sensitize the cells to chemotherapy required further characterization. In this study, we evaluated the ability of the cell-permeable Bak BH3 peptide (PETK), constituted by the Bak BH3 peptide bound to the molecular Tag Flag and to the Antennapedia fusogenic peptide, to promote apoptosis-mediated cell death and to induce chemosensitization in hematologic malignant cells.

With molecular modeling, we documented that the PETK peptidic complex is characterized by the presence of two *α*-helices connected by a loop.  Figure 1(a) shows that the Bak BH3 peptide stabilizes a small *α*-helix, as previously reported [13]. The molecular Tag Flag is part of the loop, and the Antennapedia fusogenic peptide stabilizes the major *α*-helix, also as previously described [60]. The pleating and packing reveal a type bHLH structural domain. Evaluation of its stereochemical quality showed that 99.8% of the residues are in regions in which the *φ* and *ψ* angles correspond to pleating of the *α*-helices. In terms of the theoretical prediction on the internalization of the core in the membrane, we observed that given its amino acid composition and its hydrophobicity, it is possible that it crosses the membrane through a diffusion process. With this information, the cell-permeable Bak BH3 peptide (PETK) and the PET and KT controls were synthesized.

In order to analyze the effect of the cell-permeable Bak BH3 peptide on the viability of cell lines obtained from hematologic malignancies, Ramos cells from Burkitt's lymphoma, an aggressive human B non-Hodgkin's lymphoma expressing the Bax, Bak, Bcl-_XL_, and Mcl-1 proteins but not Bcl-2 (Figure 1(b)) and CCRF-CEM cells originating from human T-cell acute lymphoblastic leukemia and expressing Bax, Bak, and Bcl-_XL_ and slightly expressing Bcl-2 and Mcl-1 also (Figure 1(b)), were treated with 20 *μ*M of the different peptides and controls. As seen in Figure 2(a), treatment with the cell-permeable Bak BH3 peptide induced death in 38% of Ramos cells, 13% more than in the positive control, CDDP (a drug used in the treatment of chemotherapy-refractory hematologic neoplasias) [45, 46]. As expected, treatment with the KT peptide that does not have the PE peptide to enter the cell showed cell death baseline values; surprisingly, in CCRF-CEM cells, we observed 74% cell death, 30% more than that observed with the positive control, CDDP (44%). Assays with active caspase-3, conducted on Ramos and CCRF-CEM cells treated with the different peptides, confirmed the cell death induced by the cell-permeable Bak peptide and is due to the restoration of apoptosis mechanisms. Therefore, in Ramos cells, apoptosis mediated by the cell-permeable Bak peptide induced a four-fold increase in active caspase-3 positive cells than those that were only treated with the KT peptide and other controls (Figure 2(b)). CCRF-CEM cells were more sensitive to the effect of the cell-permeable Bak BH3 peptide compared with the KT peptide and yielded 7 times more active caspase-3 positive cells than those treated only with the KT peptide and other controls. This increased sensitivity to treatment with the cell-permeable Bak BH3 peptide in this cell line may be explained by the low expression of the antiapoptotic molecules Mcl-1 and Bcl-2 and proapoptotic Bax protein [61] (Figure 1(b)); unlike Ramos cells, CCRF-CEM cells express the Bcl-2 protein; but in pediatric patients with ALL, its presence has been shown to not condition therapeutic drug resistance [62, 63]. In this differential activity of the cell-permeable Bak BH3 peptide among the Ramos and CCRF-CEM cells, the fusogenic peptide also could affect the outcome, since it has been reported that the Antennapedia peptide is more efficiently imported into human primary T lymphocyte compared with human primary B lymphocyte [64]. However, these results are consistent with previous studies that used the cell-permeable Bak BH3 peptide to restore apoptosis in head and neck squamous cell carcinoma cells and in T-cell acute leukemia cells that overexpress antiapoptotic molecules belonging to the Bcl-2 family [28].

The administration of subtoxic drug doses in conjunction with molecules that sensitize cells to die by apoptosis and in particular, BH3-mimetic drugs, is one of the useful strategies to decrease side effects and improve results in patients with hematologic malignancies [15]. Accordingly, we analyzed the ability of the cell-permeable Bak BH3 peptide (PETK) to induce chemosensitivity, particularly to cisplatin (CDDP), a drug used as alternative treatment in cases of refractory hematologic malignancies [45, 46]. Results in Figure 3 confirm that treatment of Ramos cells with PETK in combination with CDDP induced 62% of cell death and 39% apoptosis, compared with only PETK therapy (38% cell death and 20% apoptosis) and only CDDP (24% cell death and 14% apoptosis). These values were even greater in the chemosensitization observed in CCRF-CEM cells, in which the combination of PETK and CDDP led to 80% cell death and 64% apoptosis, compared with treatment with only PETK (64% viability and 45% apoptosis) and only CDDP (44% cell death and 26% apoptosis). These data are consistent with the increased antitumor activity of ABT-737 and its orally bioavailable derivative, navitoclax, when combined with different chemotherapeutic agents (dexamethasone, etoposide, fludarabine, vincristine, and doxorubicin among others) and used in the treatment of different tumor cell lines [56], including chemotherapy-resistant leukemias [65, 66] and lymphomas [67]. In the same context, venetoclax, an orally bioavailable Bcl-2-specific BH3 mimetic, has shown greater efficacy when combined with chemotherapies such as CHOP, R-CHOP, or bendamustine among others, in patients with relapsed or refractory non-Hodgkin's lymphoma [66, 68]. However, these encouraging results with synthetic BH3 mimetics, such as venetoclax, are often associated with severe decrease in lymphocyte counts and tumor lysis syndrome, a risk that has been mitigated by dose deescalation [15, 55, 69].

Since the Bak BH3 peptide has mostly been reported to bind Bcl-_XL_, Mcl-1, and A1 antiapoptotic proteins and that the Bax BH3 peptide binds to Bcl-2, Bcl-w, and A1 antiapoptotic proteins [70], we decided to combine the treatment with both BH3 peptides to better antagonize the complete set of antiapoptotic proteins overexpressed in hematologic malignant cells [71, 72]. Accordingly, Ramos and CCRF-CEM cells were treated with different peptides at 20 *μ*M, and the results shown in Figure 4(a) reveal that cell-permeable Bax BH3 peptide was more potent to reduce the viability compared with the cell-permeable Bak BH3 peptide in both cell lines tested. Since the PEX, used in this study, does not have the Flag peptide included in the PETK, the differences in the design could suggest that the cell-permeable Bak BH3 peptide should be designed in a better manner to enhance its activity. However, it has been reported that the cell-permeable Bax BH3 peptide is also more potent to induce death of head and neck squamous carcinoma cells comparing with cell-permeable Bak peptide, even though when both cell-permeable peptides had the same design (Antennapedia peptide in the amino-terminal domain, without the flag sequence) [28].  Figure 4(a) also reveals a surprising synergistic effect when combining the cell-permeable BH3 peptides PETK and PEX that induced cell death in 79% of Ramos cells and in 90% of CCFR-CEM cells; this cell death percentage is even greater than that obtained with the combination of the cell-permeable BH3 peptides with CDDP in previously described experiments. These data are consistent with previous studies showing that the combination of the BH3 peptides from BH3-only proapoptotic proteins act synergistically in the induction of apoptosis in neuroblastoma cells [73] and in chronic lymphocytic leukemia [74]. Similar results have been obtained with combinations of BH3 mimetic drugs in hematologic malignances [75–80]. Notably, the use of the PETK and PEX peptides induced slight toxicity on PBMC (Figure 4(b)). This reduced toxicity on PBMC is consistent with previous observations claiming that tumor cells have more sensitivity to an apoptotic stimulus comparing with normal cells, due to the “Mitochondrial priming” [81, 82], which means the proximity of a cell to the apoptosis threshold, a condition that is regulated by the balance of the antiapoptotic and proapoptotic proteins of the Bcl-2 family. Mitochondrial priming is relative, and the more primed a cell becomes, the lower the apoptotic threshold and tolerance for apoptotic stimuli. In this context, cancers are highly primed compared with the normal cells [82, 83], as has been determined by the BH3 profiling, an assay that measure the proximity of a cell to the apoptosis threshold, and also identifies the specific prosurvival proteins on which a cell depends for its survival [84].

Despite these encouraging results, it is important to mention that PETK and PEX combination treatment led to decreased viability of PBMCs at comparable levels to CDDP treatment. Although this effect was less impressive compared to lymphoma and leukemia cells, it could have implications in the therapeutic window of the peptides, as was observed for the dual Bcl-2/Bcl-_XL_ inhibitor ABT-737 and its orally bioavailable equivalent ABT-263 (Navitoclax), that showed thrombocytopenia due to inhibition of Bcl-_XL_ [84]. However, it has been also reported that the first selective and highly potent Bcl-2 inhibitor, venetoclax (formerly ABT-199/GDC-0199), induced significantly less thrombocytopenia than navitoclax [44], although this BH3 mimetic showed significant toxicities on normal human B cells as their malignant counterpart [80]. In this context, a further characterization is needed with the PETK and PEX combination treatment, to elucidate the complete effect over normal cells; for instance, perform a BH3 profile determination or make dose reductions in our assays, since our results in the Figure 5 showed that less amount on the PETK and PEX combinations (20 *μ*M and 5 *μ*M, respectively) still induced a significant reduction in the viability of the Ramos and CCFR-CEM cells.

Finally, we decided to determine whether the combination of the PETK and PEX peptides that inhibit several antiapoptotic proteins induced greater chemosensitization than that observed with a single peptide. Based on that aim, Ramos and CCRF-CEM cells were treated for 6 hrs with the PET, KT, and PETK peptides at 20 *μ*M and with the X and PEX peptides at 5 *μ*M (the peptide dose was decreased to underscore the synergistic effect on the induction of cell death in tumor cells), in the presence and in the absence of subtoxic doses of vincristine 80 nM, a commonly used drug in the treatment of hematological malignancies [50].  Figure 5 shows that the combination of the cell-permeable Bak BH3 peptide (PETK) with the cell-permeable Bax BH3 peptide (PEX) eliminated 51% of the Ramos cells and 68% of the CCRF-CEM cells; as expected, in the presence of vincristine, cell death increased significantly to 71% in Ramos cells and 82% in CCRF-CEM cells. These results suggest that treatment with the BH3 peptide combination has great potential in the chemosensitization of hematologic malignant cells, as has been shown with the BH3 mimetic drugs [15], and the use of subtoxic doses of vincristine with prior sensitization with BH3 peptide combinations would solve the problem of neurotoxicity associated with its administration in large doses in patients with hematologic malignancies [85].

## 5. Conclusions

Overall, our findings have shown the ability of the Bak BH3 peptide coupled with the Antennapedia fusogenic peptide (cell-permeable Bak BH3 peptide) to restore apoptosis and induce chemosensitization in acute lymphoblastic leukemia and non-Hodgkin's lymphoma cell lines, which may be further enhanced with the addition of the cell-permeable Bax BH3 peptide. These results represent an attractive approach to the improvement in patient outcomes, in cases of relapsed or refractory hematological malignant cells.

## Data Availability

The data used to support the findings of this study are available from the corresponding author upon reasonable request.
